# Profiled support vector machines for antisense oligonucleotide efficacy prediction

**DOI:** 10.1186/1471-2105-5-135

**Published:** 2004-09-22

**Authors:** Gustavo Camps-Valls, Alistair M Chalk, Antonio J Serrano-López, José D Martín-Guerrero, Erik LL Sonnhammer

**Affiliations:** 1 Grup de Processament Digital de Senyals, Universitat de València, Spain. C/ Dr. Moliner, 50. 46100 Burjassot, València, Spain; 2 Center for Genomics and Bioinformatics (CGB), Karolinska Institutet, S-17177, Stockholm, Sweden

## Abstract

**Background:**

This paper presents the use of Support Vector Machines (SVMs) for prediction and analysis of antisense oligonucleotide (AO) efficacy. The collected database comprises 315 AO molecules including 68 features each, inducing a problem well-suited to SVMs. The task of feature selection is crucial given the presence of noisy or redundant features, and the well-known problem of the *curse of dimensionality*. We propose a two-stage strategy to develop an optimal model: (1) feature selection using correlation analysis, mutual information, and SVM-based recursive feature elimination (SVM-RFE), and (2) AO prediction using standard and profiled SVM formulations. A profiled SVM gives different weights to different parts of the training data to focus the training on the most important regions.

**Results:**

In the first stage, the SVM-RFE technique was most efficient and robust in the presence of low number of samples and high input space dimension. This method yielded an optimal subset of 14 representative features, which were all related to energy and sequence motifs. The second stage evaluated the performance of the predictors (overall correlation coefficient between observed and predicted efficacy, *r*; mean error, ME; and root-mean-square-error, RMSE) using 8-fold and minus-one-RNA cross-validation methods. The profiled SVM produced the best results (*r *= 0.44, ME = 0.022, and RMSE= 0.278) and predicted high (>75% inhibition of gene expression) and low efficacy (<25%) AOs with a success rate of 83.3% and 82.9%, respectively, which is better than by previous approaches. A web server for AO prediction is available online at .

**Conclusions:**

The SVM approach is well suited to the AO prediction problem, and yields a prediction accuracy superior to previous methods. The profiled SVM was found to perform better than the standard SVM, suggesting that it could lead to improvements in other prediction problems as well.

## Background

The expression of a gene can be inhibited by antisense oligonucleotides (AOs) targeting the mRNA. However, if the target site in the mRNA is picked randomly, typically 20% or less of the AOs are effective inhibitors *in vivo *[1]. The sequence properties that make an AO effective are not well understood, thus many AOs need to be tested to find good inhibitors, which is time-consuming and costly.

Antisense oligonucleotides contain 10–30 nucleotides complementary to a specific subsequence of an mRNA target, which are designed to bind to targets by standard Watson-Crick base pairing rules. The bound duplex can knockdown gene expression through a number of mechanisms. These are RNase-H mediated cleavage, inteference with translation or splicing and destabilization of the target mRNA [2-4]. The AO inhibits gene expression in a specific and reversible manner, a process termed 'Gene knock-down' and all mechanisms leave the AO intact to induce further knock-down. For a comprehensive review of the topic see [5].

There are many laboratory-based strategies for selecting AOs. A classical approach is the 'gene-walk' approach, in which 15 or more AOs are evaluated for a gene in order to find a sufficiently effective AO. Methods with higher reliability experimentally determine mRNA regions that are accessible to RNase-H clevage and therefore more likely to be an e'ective site for AOs [6-8]. In general, the experimental approaches are time consuming and expensive.

There are many examples in the literature of experimental groups attempting to correlate AO sequence properties with efficacy. A correlation between binding energy (AO-RNA) and efficacy has been observed [6,9]. Particular target secondary structures have been shown to correlate with efficacy [10]. However, the correlations are not consistently detected across studies. This variation can be due to many factors including biases in the selection of the AOs, varying experimental conditions, or, in cases where computational RNA folding prediction was used, limitations in the structure prediction methods. In [11], published AOs were examined and recomended values for dimer, hairpin and ΔG to increase the proportion of higher efficacy AOs were given.

AO selection can be based on either experimental or theoretical approaches (for a review, see [12]). Computational approaches to AO design have so far focused on prediction of the structure of the target mRNA and from this deriving the accessibility of target regions (e.g. [12-19]). Perhaps the most successful method is that of Ding and Lawrence [19], using a statistical sampling of secondary structures to predict accessible regions to find effective AOs for rabbit *β*-globin. In general, methods have not been evaluated on a broad range of gene targets. Another method is to look for motifs that occur more often in effective AOs. Ten sequence motifs have been identified with a correlation to AO efficacy in [20], and recently, motifs have been used as the input to neural network models [21,22] with reasonable success.

In this context, the challenge is hence to discover general principles that hold across all AO studies. One approach to discover such principles is to explore a diverse range of sequence properties and incorporate the factors that affect AO efficacy into a computational model for AO design. This requires both a database of tested AOs, such as that produced by [21,23], and machine learning methods of model building. The database should be based on large AO screening experiments to ensure comparability. In this context, the use of advanced pattern recognition methods such as neural networks or Support Vector Machines (SVMs) is becoming very popular because of their good capabilities for classification, function approximation and knowledge discovery. In particular, the use of SVMs in bioinformatics has found a natural match because they work efficiently with high input dimension spaces and low number of labeled examples. As a consequence, many biological problems have been solved in this field. The interested reader can visit [24] for a collection of SVM applications in bioinformatics. However, the use of the SVM has been traditionally attached to the classification problem, and few efforts have been made to tackle the regression (or function approximation) problem.

This paper proposes the use of SVMs for prediction and analysis of AO efficacy. The collected database comprises 315 AO molecules including 68 features each, which induces *a priori *a well-suited problem to SVMs, given the low number of samples and high input space dimension [25]. Nevertheless, the problem of feature selection becomes crucial because the number of examples in the database (AO molecules) is low compared to the number of features for each of them and, therefore, overfitting is likely to occur, reducing the performance of the model [26,27]. Additionally, being able to explain the obtained solution (in terms of the selected input features) can be as relevant as obtaining the best possible predictor. This is of particular interest in bioinformatics in general and for AO efficacy prediction in particular, as was previously illustrated in [21,22]. The issue of feature selection in the SVM framework has received attention in the recent years [28-32]. The fact that SVMs are not *drastically *affected by the input space dimensionality has sometimes led to the wrong idea that a feature selection is not necessary at all. The guiding principle of SVMs ensures certain robustness to outliers or abnormal samples in the distribution inherently, but the selection of the optimal subset of features is still an unsolved problem in the literature. We can state that in most applications, the success of machine learning is strongly affected by data quality (redundant, noisy or unreliable information) and thus a feature selection is not only recommendable but mandatory.

In this paper, we propose a two-stage strategy to tackle the problem:

1. *Feature selection. *This task is carried out using three techniques: correlation analysis, the mutual information feature selection (MIFS) method, and the SVM-based recursive feature elimination (SVM-RFE).

2. *AO efficacy prediction. *We develop standard and profiled SVMs to accomplish this task. Several measures of accuracy of the estimations and two cross-validation methods are used in order to attain both significant and robust results.

## Methods

### Data collection

In the present work, we have extended the database used in [21] by including 68 features for each AO. The so-called AO database (AOdb) was assembled from a selection of AO publications. Published data was incorporated for which: (a) at least 6 AOs were tested under the same experimental conditions, although more than one gene target were allowed; (b) efficacy of the AOs were presented as a percentage of the control level of the target gene expression, either as RNA or protein. No papers were reported matching these criteria before 1990, as is consistent with [23]. Accompanying this data is the full RNA sequence and accession number (where available) together with positional coordinates of the AOs and the position of the coding sequence. Publication details, cell line used and the chemistry of the AOs were also recorded in the database. The database consists of 315 oligonucleotides from 15 studies testing AO efficacy on 13 genes. The essential information in the database is AO sequence and efficacy expressed as (100% - [% of control expression])/100. For the cases where the same AO is tested in two different laboratories, or twice by the same laboratory the average efficacy is used.

A set of *a priori *representative parameters was derived from the information contained in the AO sequence collection, including values for: (1) base composition (Number of A/C/G/T, % GC content): (2) RNA-AO binding properties (binding energy, enthalpy, entropy): (3) RNA-AO terminal properties (3' binding energy, 5' binding energy); (4) AO-AO binding properties (Hairpin energy and quality, Dimer energy); and (5) 9 of the 10 verified sequence motifs correlated with efficacy from [20]. Binding energy calculations were completed using thermodynamic parameters from [33]. The calculation of dimer energy was made using an ungapped alignment with stacking energies taken from [34] and a uniform penalty 0.5 for mismatches. Hairpin energy was calculated using both Mfold [35] and the Vienna package [36]. Parameters describing cellular uptake and protein interactions were not included, as we have no explicit way of modeling them. A number of additional features were included to complete the AOdb: motifs, AO position, predicted conformation of the target structure, single-strandedness, binding energies from [14]. For brevity, the complete list and more information on the database can be obtained at [37]. The database is available under request.

### The feature selection problem

The Feature Selection Problem (FSP) in a "learning from samples" approach can be defined as choosing a subset of features that achieves the lowest error according to a certain loss functional [28]. Following a general taxonomy, the FSP can be tackled using *filter *[38] and *wrapper *[26] methods. Filter methods use an indirect measure of the quality of the selected features, e.g. evaluating the correlation function between each input feature and the observed output. A faster convergence of the algorithm is thus obtained. On the other hand, wrapper methods use as selection criteria the goodness-of-fit between the inputs and the output provided by the learning machine under consideration, e.g. a neural network. This approach guarantees that, in each step of the algorithm, the selected subset improves performance of the previous one. Filter methods might fail to select the right subset of features if the used criterium deviates from the one used for training the learning machine, whereas wrapper methods can be computationally intensive due to the learning machine has to be retrained for each new set of features. In this paper, we evaluate the performance of SVMs for different subsets of relevant features, which are selected using both filter and wrapper approaches.

### Correlation analysis and mutual information

A common practice to evaluate the (linear) relationship between each of the *n *input features 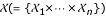 and output 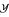, or among pair-wise inputs (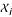 and 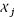) is the use of the correlation function. This is a good method to remove redundant features and to evaluate relationships, but fails when working with low number of samples, or when the assumed linear relationship is not present. When data is considered as the realization of random processes, it is possible to compute the relevance of variables with respect to each other by means of the mutual information (MI) function, which is defined as the difference between entropy of 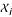 and the conditional entropy of 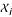 given 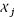. The MI function is suitable for assessing the information content of features in tasks where methods like the correlation are prone to mistakes. In fact, the MI function measures a general dependence between features, instead of a linear dependence offered by the correlation function. In [39], an algorithm called Mutual Information Feature Selection (MIFS) was successfully presented. The method greedily constructs the set of features with high mutual information with the output while trying to minimize the mutual information among chosen features. Thus, the *ith *input feature 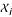 included in the set, maximizes 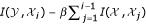 over all remaining features 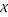 for some parameter *β *∈ (0,1]. The feature selection procedure is performed iteratively until a desired number of features is reached. We will use the correlation function and the MIFS method as filter methods, i.e. a feature ranking will be provided and only the most important features will be accounted for modeling.

### Support vector regressor (SVR)

Support Vector Machines are state-of-the-art tools for nonlinear input-output knowledge discovery [40]. The Support Vector Regressor (SVR) is its implementation for regression and function approximation, which has been used in time series prediction with good results [41]. Basically, the solution offered by the SVR takes the form 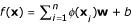, where **x**_*i *_is an input example, *φ *is a nonlinear mapping, **w **is a weight vector and *b *is the bias of the regression function. In the SVR, a fixed desired accuracy *ε *is specified a priori and thus one tries to fit a "tube" with radius *ε *to the training data. The standard SVR tries to minimize two factors: the norm of the squared weight vector, ||**w**||^2^, and the sum of permitted errors. These two factors are traded-off by using a fixed penalization parameter, *C*. We can formally state the SVR method as follows: given a labeled training data set {(**x**_*i*_, *y*_*i*_), *i *= 1,..., *n*}, where **x**_*i *_∈ ℝ^*d *^and *y*_*i *_∈ ℝ, and a nonlinear mapping to a higher dimensional space *φ*: ℝ^*d *^→ ℝ^*H *^where *d *≤ *H*, find the minimum of the following functional with respect to **w**, *ξ*_*i*_, 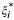 and *b*:


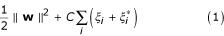


subject to:


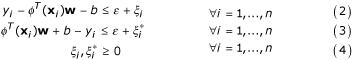


where 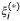 and *C *are, respectively, positive slack variables to deal training samples with a prediction error larger than *ε *(*ε *> 0) and the penalization applied to these ones. These two parameters are tuned by the user.

The usual procedure for solving the SVR introduces the linear restrictions (2)-(4) into (1) by means of Lagrange multipliers *α*_*i *_and 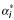 associated to each constraint. The dual functional obtained has to be minimized with respect to primal variables (**w**, *ξ*_*i *_and 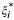) and maximized with respect to dual variables (*α*_*i*_). The optimization of the obtained dual problem is usually solved through quadratic programming procedures [40,42], and the final solution provided by the SVR for a test example **x **can be expressed as


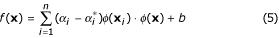


where only the non-zero Lagrange multipliers account in the solution. The corresponding input examples are called *support vectors *and represent the most critical samples in the distribution.

An important characteristic of the SVR training methodology is that one does not need to know explicitly the form of the mapping *φ *(**x**) but only a kernel function, which maps the samples into a high dimensional space. This kernel function appears in the form of dot products in (5), *K *(**x**_*i*_, **x**_*j*_) = *φ*(**x**_*i*_)·*φ*(**x**_*j*_) and can be viewed as a measure of similarity between samples. Therefore, in order to train the SVR model, one only has to select a kernel function, its free parameters, the parameter *C*, and the size of the *ε*-insensitivity zone. In this paper, we have only used the Gaussian (or Radial Basis Function, RBF) kernel, given by:

*K *(**x**_*i*_, **x**_*j*_) = exp (-*γ*||**x**_*i *_- **x**_*j*_||^2^).     (6)

There are some reasons to select the RBF kernel *a priori*. The RBF kernel maps samples into a higher dimensional space so, unlike the linear kernel, it can handle efficiently cases in which the relation between the dependent and independent variables is non-linear. The RBF kernel has less numerical difficulties than sigmoid or linear kernels. In fact, sigmoid kernels behave like RBF for certain parameters [43,44] but unfortunately, they are non-positive definite kernels in all situations, which precludes their practical application [25]. Finally, for using the RBF kernel, only the Gaussian width has to be tuned. For tutorials, publications, and software resources on SVM and kernel-based methods, the reader can visit [45].

### Recursive feature elimination (SVM-RFE)

The SVM-RFE algorithm has been recently proposed in [29] for selecting genes that are relevant for a cancer classification problem. The goal is to find a subset of size *m *among *n *features (*m *<*n*) that maximizes the performance of the predictor for a given measure of accuracy. This is a wrapper method and involves high computational cost. The method is based on a backward sequential selection. One starts with all the features and removes one feature at a time until *m *features are left. Basically, in each iteration, one focuses on the weight vector, which constitutes the solution provided by the SVR and therefore, its analysis is of fundamental relevance to understand the importance of each input feature. The removed feature is the one whose removal minimizes the variation of ||**W**||^2^. Hence, the ranking criterion *R*_*c *_for a given feature *i *is:


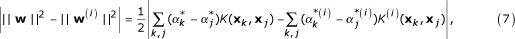


where *K*^(*i*) ^is the kernel matrix of training data when feature *i *is removed 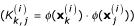 and 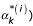 are the Lagrange multipliers corresponding to sample *k *when the input feature *i *is removed. The idea underlying this procedure is basically to evaluate at each iteration which feature affects less the weight vector norm and, consequently, to remove it.

## Results

In this section, we present and discuss the results obtained both regarding feature selection and prediction accuracy. Filter and wrapper feature selection methods will provide different subsets of representative features. SVMs are trained for each subset and their performance is evaluated in terms of robustness and accuracy.

### Feature selection

The first approach to the FSP consisted of performing a correlation analysis in order to identify redundant variables. We adopted a similar strategy followed in [21], i.e. to remove features correlated to each other at >0.9 (*p *< 0.001), keeping the highest correlation to efficacy. This analysis discarded 12 redundant features out of the 68 original ones, and additionally provided a ranking of the most correlated features to efficacy. We finally selected the 14 top ranked features according to this criterion, ranging in correlation from -0.35 (ΔG) to -0.16 (# Adenine). We selected this number of features for the purpose of a fair comparison with the best subset in [21]. Table 1 shows selected features in both cases. Note that some di'erences are observed between the present work and [21] with regards the value of the correlation coefficient, 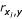 (first and last columns, respectively). They are due to the facts that (1) we have included here very low efficacy oligos in the calculation, and that (2) because more features were added to the AO database, e.g. predicted secondary structure, oligos had to be discarded when the target RNA was unavailable.

A feature ranking according to the correlation coefficient can be useful to analyze input-output linear dependencies, but it is not good practice to rely only on this decision to build a model. As a second approach, we ran the MIFS method and selected a desired subset of best 14 features. We selected *β *= 0.75, which yielded a balanced estimation of both the MI with the output (AO efficacy), and the already-selected features. The more important features match the ones selected using the correlation function, but MIFS also included hairpin measurements. This is due to the fact that MIFS is not based on correlatedness but on mutual dependence criteria.

A third approach was the use of SVMs based on the RFE method. In this task, we trained an SVM to predict AO efficacy using all available features. It should be noted here that RFE is a wrapper method that involves a very high computational burden since the SVM must be retrained in each iteration with the selected features. The best model was selected by evaluating the RMSE (accuracy of the estimations) in the validation set through the 8-fold cross-validation method, which splits the data into eight parts, and uses seven parts for training and the eighth one for validation. The procedure is then repeated eight times. In our implementation, we included the possibility suggested in [29] by which it is possible to remove chunks of features at each iteration –a maximum value around 10 was a suitable option. In our application, only ten iterations were necessary to achieve the best 14 features (see Table 1). In [20,21], a surprising lack of correlation was observed between dimer energy and efficacy, which was attributed to some kind of bias in the databases. In the present work, nevertheless, SVM-RFE includes dimer energy as the 11th most relevant feature. In conclusion, SVM-RFE selects a combination of highly correlated but also mutually informative features.

We can also conclude that noticeable differences are observed between the obtained rankings. A possible explanation for discrepancies of this sort is the non-linear mapping that SVR methods perform. Explaining those input-output relationships is often difficult and biased conclusions are usually obtained. Different families of methods (SVM, neurofuzzy, decision trees, or neural networks) perform different mappings due to their specific guiding principles (structural risk minimization, membership optimization, entropy-based criteria, or empirical risk minimization, respectively) and thus, the interpretation of these methods is quite diffcult. In addition, different models (topologies, structures, kernels, membership functions) in a family would surely yield different results.

### Model development

A greedy search was carried out for the free parameters (*C*, *ε*, *γ*) As regards the penalization parameter, it is a common practice trying exponentially increase sequences of *C *(*C *= 10^-2^, 10^-1^,..., 10^3^). In our case study, we achieved good results in the range of *C *∈ [1,1000]. The insensitivity zone was varied linearly in the range [0.001, 0.3]. The *γ *parameter was exponentially varied in the range *γ *= 10^-7^,...,10^-1^. For each free parameter combination, we evaluated the performance of the predictors through several measurements: the correlation coefficient between actual and predicted efficacies (*r*), the mean error (ME), and the root-mean-square-error (RMSE). Additionally, we computed the rate of observed efficacies above a defined predicted threshold of 0.75 (SR_>0.75_) and below 0.25 (SR_<0.25_). These prediction ranges are of particular interest, since they stand for high and low AO efficacies, respectively. In fact, it is not only important to identify high efficacy oligos but also factors causing AOs to be completely ineffective ([0,0.25]). However, care must be taken as more noise can be present in the low efficacy region.

### Model comparison

At the first stage of the work, we trained SVMs using the 8-fold cross-validation method for RFE-based feature selection. However, this training methodology can lead to overoptimistic results because AOs on the same gene are not always independent data points. Hence, we also followed a different strategy, which entails removing all AOs targeting one gene for training, training the model, and then testing performance on predicting the efficacy of these oligos. This is a common method [22] and we refer to it as minus-one-RNA cross-validation (-RNA). It safely removes any overlap between training and test data, and thus ensures the generality of the model.

**Figure 1:**
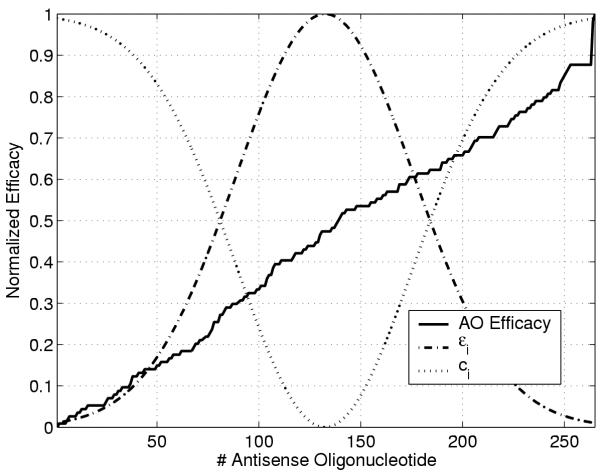
Illustration of Gaussian-like profiles for the penalization factor and the *ε*-insensitive region in the P-SVR approach. In this case, we penalize harder the committed errors in the higher and lower efficacy regions. Additionally, the insensitive region becomes wider in medium AO efficacies, and thus few AOs will contribute to the cost function and, consequently, become support vectors. Only one additional parameter is introduced in the formulation, i.e. the width of the Gaussian profile, *σ*_*P*_.

In AO prediction, we are most interested in predicting good oligos (high efficacy, > 0.75), and those that are bad (low efficacy, < 0.25). This previous knowledge about the problem can be introduced in the SVM formulation by tailoring specific *confidence functions *for the adaptation of the penalization factor *C*, and the *ε*-insensitive zone of each sample. The so-called Profiled SVR (P-SVR) [46] obviously implies making some changes in the original SVR formulation since now *C *and *ε *become sample-dependent. In [46,47], we designed profiles for the variation of *C *and *ε *in complex pharmacokinetic problems. In this paper, our intention relaxing or tightening *ε *and *C *depending on the observed AO efficacy value. A proposal for this variation is illustrated in figure 1. Note that we increase the penalization of errors committed in the high or low AO efficacy ranges since we are more interested in obtaining good results in these regions.

Additionally, the *ε*-insensitivity zone is reduced in these regions thus forcing a reduced error there. Some other profiles could be introduced in the training methodology without loss of generality.

Results for all approaches are shown in Table 2 for the validation set. We observe that RFE is the best method for selecting features. The choice of cross-validation method does not make much difference; the RMSE is the same while the goodness-of-fit (*r*) is almost unchanged. Using the P-SVR method (with features selected by the 8-fold crossvalidated RFE) we gained substantially in RMSE, and also obtained a better balance between the success rates of high and low predictions. This indicates that the P-SVR improves the performance of standard SVR even without a dedicated feature selection method, and suggests that even better results could be obtained if P-SVR were embedded in the RFE feature selection procedure.

These outcomes are worth analyzing because one could expect worse results when using -RNA cross-validation since this method removes the possibility of cross-talk in the training phase between overlapping oligos. However, we have to stress here that, by training the SVR with -RNA cross-validation, one only improves the *r *indicator, which is a biased estimator of the accuracy. In fact, accuracy (RMSE) remains basically the same, and bias (ME) becomes positive and higher, which could induce some distrust for the model. When analyzing results from the P-SVR, we can observe a general improvement in all indicators, which is basically due to the fact that by tightening the "tube" around the interesting ranges, a higher number of support vectors is selected there (but lower in the overall domain), which induces a richer solution in the interesting zones. In addition, the profiled *C *parameter penalizes higher the committed errors in these zones, which is particularly interesting to deal with outlying samples in the distribution and to provide a smoother solution in these particular zones. The designed profile, nevertheless, could lead to an overfitted solution in the interesting zone if *ε*_*i *_and *C*_*i *_were not well-controlled. However, by using the-RNA cross-validation method, this threat is avoided and better results are finally obtained. Therefore, the combined strategy of P-SVR and -RNA cross-validation results in a balanced and robust predictor. Additional consequences can be extracted: (1) the correlation coefficient is relatively low for all methods but superior to the ones obtained in [21]; (2) differences among the models are neither numerically (see Table 2) nor statistically significant as tested with One Way Analysis-Of-Variance (ANOVA) in bias (*F *= 0.01, *p *= 0.811) or accuracy (*F *= 0.06, *p *= 0.567); (3) prediction is more accurate, in general terms, for the higher efficacy levels (SR_>0.75_, > SR_<0.25_), as also noted in [22]; and (4) SVM-RFE can deal efficiently with high input spaces and produces robust results (compare results with those from the "All features" subset). Additionally, we can conclude that the P-SVR improved results in terms of accuracy of the predictions compared to the standard SVR.

## Conclusions

In this paper, we have used standard and state-of-the-art methods for knowledge discovery in a relevant bioinformatics problem: the analysis and prediction of AO efficacy. We have engineered robust and accurate SVMs, and used filter and wrapper feature selection methods in order to build representative subsets of input features. Compared to [21], our results represent a significant improvement. In that work, SR_>0.8 _was reported to be 50%, and *r *= 0.30. The success of the P-SVR for the AO prediction problem suggests that it could be successfully applied to other prediction problems. A web server for AO prediction is available online at [48].

Our future work is concentrated to improving results with more careful design of profiles by the inclusion of fuzzy and rough sets. Additionally, we are exploring the possibility of providing confidence values for the predictions in the form of *p*-values from the Lagrange multipliers. This way, the user could get a set of best predictions back, then a second set that is more likely to be less accurate, and so on. This would allow the lab-user to choose the best ones first, but if they fail specificity controls they would have another set to work with.

## Authors' contributions

GCV carried out the training of the feature selection and regression methods. AC participated in model development and testing process, and developed the web-server. AJSL collaborated in model development and assessment. JDMG engineered the profile function. ES conceived and coordinated the study. All authors contributed to the manuscript preparation, and approved the final manuscript.

## Figures and Tables

**Table 1:** Feature ranking using (a) the correlation coefficient between input features and efficacy (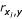), (b) mutual information feature selection (MIFS) with *β *= 0.75, (c) SVM-based Recursive Feature Elimination (SVM-RFE), and (d) best selection in [21] using the correlation coefficient.

	**FEATURE**	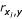	**FEATURE**	**MI **(*β *= 0.75)	**FEATURE**	**SVM-RFE **||**W**||^2^	**FEATURE**	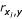 **in [21]**
1	ΔG	-0.35	ΔG	0.094	ΔH	0.680	GGGA	0.26
2	# Cytosine	0.31	# Cytosine	0.089	ΔS	0.671	# Cytosine	0.23
3	TCCC	0.28	%GC content	0.077	ΔG	0.193	ΔH	-0.19
4	5pΔG	-0.26	ΔG/length	0.075	# Cytosine	0.045	ΔG	-0.18
5	ΔH	-0.24	ΔH	0.064	Hairpin quality	0.035	CAGT	-0.18
6	ΔH/length	-0.22	ΔH/length	0.061	# Adenine	0.024	AGAG	0.18
7	%GC content	0.22	ΔS	0.060	# Thymine	0.018	GTGG	0.17
8	CCCT	0.21	# Adenine	0.043	Hairpin length	0.014	# Guanine	-0.15
9	CCAC	0.21	# Guanine	0.042	5pΔG	0.009	3pΔG	0.14
10	CCCC	0.21	5pΔG	0.040	3pΔG	0.005	ΔS	-0.14
11	CTCT	0.20	Hairpin quality	0.027	Dimer	0.004	CCCC	-0.13
12	CCCA	0.20	Hairpin length	0.024	Hairpin energy (Mfold)	0.003	Hairpin quality	-0.11
13	ACAC	-0.16	Hairpin Energy	0.022	# Guanine	0.001	%GC content	0.11
14	# Adenine	-0.16	# Thymine	0.016	Hairpin energy (vienna)	0.000	TGGC	-0.10

**Table 2:** Mean error (ME), root-mean-squared error (RMSE) and correlation coefficient (r) of models in the validation set. Success rates (SR) for efficacy higher than 0.75 or below 0.25 are also given for each feature selection method.

**Methods**	**SVR**					**P-SVR**
**Selection**	**All feats.**	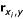	MI(*β *= 0.75)	**RFE**	**RFE**	**RFE**

**CV method**	-	-	-	**8-fold**	**-RNA**	**-RNA**

**r**	0.356	0.367	0.374	0.398	0.430	0.440
**ME**	-0.0280	-0.0223	-0.0104	-0.0068	0.031	0.022
**RMSE**	0.312	0.300	0.301	0.299	0.299	0.278
**SR**_>0.75_	82.8	87.5	86.7	87.5	83.3	83.3
**SR**_<0.25_	71.4	73.9	71.4	73.9	76.2	82.9
